# Co-morbidity of depression and anxiety in common age-related eye diseases: a population-based study of 662 adults

**DOI:** 10.3389/fnagi.2013.00056

**Published:** 2013-10-02

**Authors:** Ranmalee Eramudugolla, Joanne Wood, Kaarin J. Anstey

**Affiliations:** ^1^Centre for Research on Ageing Health and Wellbeing, The Australian National UniversityCanberra, ACT, Australia; ^2^Faculty of Health, School of Optometry and Vision Science, Queensland University of Technology, Kelvin GroveBrisbane, QLD, Australia

**Keywords:** aging, vision disorders, sensory impairment, depression, anxiety, epidemiology

## Abstract

This study examined the prevalence of co-morbid age-related eye disease and symptoms of depression and anxiety in late life, and the relative roles of visual function and disease in explaining symptoms of depression and anxiety. A community-based sample of 662 individuals aged over 70 years was recruited through the electoral roll. Vision was measured using a battery of tests including high and low contrast visual acuity, contrast sensitivity, motion sensitivity, stereoacuity, Useful Field of View, and visual fields. Depression and anxiety symptoms were measured using the Goldberg scales. The prevalence of self-reported eye disease [cataract, glaucoma, or age-related macular degeneration (AMD)] in the sample was 43.4%, with 7.7% reporting more than one form of ocular pathology. Of those with no eye disease, 3.7% had clinically significant depressive symptoms. This rate was 6.7% among cataract patients, 4.3% among those with glaucoma, and 10.5% for AMD. Generalized linear models adjusting for demographics, general health, treatment, and disability examined self-reported eye disease and visual function as correlates of depression and anxiety. Depressive symptoms were associated with cataract only, AMD, comorbid eye diseases and reduced low contrast visual acuity. Anxiety was significantly associated with self-reported cataract, and reduced low contrast visual acuity, motion sensitivity and contrast sensitivity. We found no evidence for elevated rates of depressive or anxiety symptoms associated with self-reported glaucoma. The results support previous findings of high rates of depression and anxiety in cataract and AMD, and in addition show that mood and anxiety are associated with objective measures of visual function independently of self-reported eye disease. The findings have implications for the assessment and treatment of mental health in the context of late-life visual impairment.

## Introduction

Age-related eye disease and associated visual impairment is estimated to affect over 372 million older adults globally, or 27.8% of older adults (World Health Organization, 2012), and this number is expected to rise with projected increases in the aging population (Ruiz-Moreno et al., 2007). Visual impairment and the consequent disability and loss of quality of life in this age group significantly impacts mental health (Lotery et al., 2007). Although the rate of depression increases with age, it is 1.25–2.92 times higher for older adults with vision impairment (Hayman et al., 2007; Noran et al., 2009; Li et al., 2011), and age-related eye diseases such as cataract (Fagerstrom, 1994; McGwin et al., 2003; Freeman et al., 2009), glaucoma (Wilson et al., 2002; Mabuchi et al., 2012; Tosini et al., 2012) and macular degeneration (Brody et al., 2001; Casten et al., 2004; Tolman et al., 2005; Mathew et al., 2011). In comparison, less is known about the rates of anxiety in older adults with visual impairment (Evans et al., 2007; Li et al., 2011). In the context of eye disease, both depression and anxiety present significant impediments to patient recovery and rehabilitation (Tolman et al., 2005; Walker et al., 2006), increase levels of disability (Brody et al., 2001; Banerjee et al., 2008), and ultimately represent a social and economic health burden on the community Lotery et al., 2007; World Health Organization, 2012. Thus, a clear understanding of the contributors to depression and anxiety in older adults with visual impairment has implications for treatment and public health.

Currently, there is a lack of data comparing the rate of depression across different eye diseases in late life. Most studies that have examined depression in age-related eye diseases have either focused on a single pathology (Fagerstrom, 1994; Brody et al., 2001; McGwin et al., 2003; Freeman et al., 2009; Mathew et al., 2011; Mabuchi et al., 2012), or vision impairment without identifying the eye disease causing the visual loss (Hayman et al., 2007; Li et al., 2011), or have compared common eye diseases without adjusting for level of vision impairment (Horowitz et al., 2005; Ruiz-Moreno et al., 2007). Because the prognosis, treatment options and impact on vision vary considerably across the common eye pathologies of cataract, glaucoma and macular degeneration (Voleti and Hubschman, 2013), it is expected that patients' outlook and the rate and severity of depression will also vary with disease type. For example, age-related macular degeneration (AMD), the leading cause of irreversible visual loss in older adults, affects central vision due to damage at the macular region, and has a poorer prognosis compared to reversible eye conditions such as cataract. This loss of central vision greatly impairs hobbies, close work, reading and recognizing faces (Casten and Rovner, 2008), with significant impacts on quality of life, independence and mood (Cruess et al., 2007). In contrast, glaucoma often has an insidious onset, primarily affecting peripheral vision, such that its impact on functional activities may not be apparent until quite advanced (Wilson et al., 2002; Tosini et al., 2012; Voleti and Hubschman, 2013). Furthermore, unlike cataract and glaucoma, current treatment options for AMD are limited, and methods for slowing progression have modest success rates (Voleti and Hubschman, 2013). Consequently, much of the literature has focused on depression in AMD compared to other eye diseases.

The rate of co-morbidity of major depression and AMD is high (Brody et al., 2006; Rovner et al., 2007; Ruiz-Moreno et al., 2007; Sun et al., 2007; Banerjee et al., 2008), and up to a third of patients with AMD display sub-clinical depression. Even sub-clinical depressive symptoms in AMD are associated with levels of disability comparable to that of patients with AMD and major depression (Horowitz et al., 2005; Rovner et al., 2006). A few studies have failed to find increased rates of depression in AMD (Sun et al., 2007). However, many studies have not adjusted for the association between severity of visual impairment and depression (Horowitz et al., 2005; Ruiz-Moreno et al., 2007; Banerjee et al., 2008) or comorbid eye conditions (Rovner et al., 2006), or have used selected clinical samples that may not be representative of the general AMD population (Tolman et al., 2005; Banerjee et al., 2008). The relationship between AMD and depression is likely to be a complex interaction between the disease, visual loss, quality of life, disability and comorbidity (Hawkins, 2001; Freeman et al., 2009; Mathew et al., 2011).

While little is known about the biological links between ocular pathology and mood and anxiety, there is some evidence that reduced absorption of light due to retinal damage in AMD and also glaucoma may lead to disturbed synthesis of melatonin, which in turn increases the risk of sleep disturbance, depression, and anxiety (Tosini et al., 2012). In addition, chronic conditions such as diabetes and heart disease are known to be risk factors for the development of AMD, cataract and glaucoma (Lotery et al., 2007; Casten and Rovner, 2008) and are also independently associated with depression (Horowitz et al., 2005). Dementia is also associated with depression (Lyketsos et al., 2002) and also vision problems as a result of neuro-degeneration (Trick and Silverman, 1991). Thus, comorbid conditions, type of ARED and level of visual impairment need to be considered separately when explaining depression or anxiety in AREDs.

Depression in eye-disease has also been examined in relation to treatment. Several studies have examined the impact of cataract surgery on depression (McGwin et al., 2003; Mitsonis et al., 2006; Walker et al., 2006; Freeman et al., 2009; Meuleners et al., 2013) and anxiety (Mitsonis et al., 2006; Meuleners et al., 2013). Depressive symptoms in cataract patients awaiting surgery are higher in those with poorer visual acuity in the eye to be operated on (Freeman et al., 2009). However, whether these depressive symptoms abate after surgery is unclear (McGwin et al., 2003; Mitsonis et al., 2006). Evidence for the relationship between glaucoma and depression or anxiety is mixed, with some reports of significantly higher prevalence relative to healthy controls, and others indicating no relationship after adjusting for health conditions (Wilson et al., 2002; Tosini et al., 2012). Nevertheless, there is a suggestion that anti-glaucoma drugs may increase the risk of depression (Tosini et al., 2012; Voleti and Hubschman, 2013).

These aforementioned variations in eye disease with respect to prognosis, biology, comorbid conditions and treatment, all highlight the issue that different eye diseases may have varying links with depression and anxiety and that these inter-relationships warrant further investigation. This may enable identification of individuals at risk of poorer mental health associated with eye disease (Casten and Rovner, 2008). In the present study, we investigated the prevalence of self-reported cataract, glaucoma and AMD and their associations with depression and anxiety symptoms. We also examined whether objective measures of functionally relevant visual abilities such as visual acuity, contrast sensitivity, visual fields, and motion perception were associated with symptoms of depression and anxiety independently of self-reported ocular disease. The findings have implications for identification of at-risk older adults with specific eye disease to enable better-targeted prevention and treatments.

## Methods

### Participants

A community-based sample of 750 individuals over the age of 70 years residing in the Brisbane and Sydney metropolitan areas (Australia) was recruited through the electoral roll for a study on prevention of injury in older persons. In Australia, voting is compulsory and therefore, all members of the community who are eligible to vote are registered on the electoral roll. The response rates for the Brisbane and Sydney sites were 18.84 and 28.56%, respectively. Participants were recruited into the study if they were living independently without mobility aids, and were aged between 70 and 95 years. Participants were excluded if their MMSE score was below 24, suggesting cognitive impairment or dementia. Of this sample, 662 participants provided complete data on the outcome variable—the Goldberg Anxiety and Depression (GAD) scale and were included in subsequent analyses. Participants attended two laboratory-based sessions that included visual, cognitive, and sensorimotor assessments. The study received approval by the UNSW and QUT Human Research Ethics Committees.

### Measures

#### Vision tests

***Visual Acuity.*** Visual acuity was measured binocularly using a high and low contrast Bailey-Lovie logMAR chart (Bailey and Lovie, 1976) with participants' usual distance corrective lenses, where applicable, with the chart positioned at a distance of 3.2 m under standard illumination levels. Participants were instructed to guess letters, even when they were unsure, until a full line of letters was incorrectly read. Visual acuity was scored letter-by-letter and visual acuity expressed in Snellen notatation as well as the logarithm of the minimum angle of resolution (logMAR). Mild visual impairment was defined as 20/40 to 20/60 (logMAR 0.3–0.5), moderate as 20/80 to 20/160 (logMAR 0.6–0.9) and severe is 20/200 or worse (logMAR of 1.0 or greater). Give the reference for this definition.

***Melbourne Edge Test (MET).*** Contrast sensitivity was measured binocularly using the paper version of the Melbourne Edge Test (MET) (Verbaken and Johnston, 1986) at a working distance of 40 cm and standard illumination levels. Participants wore their corrective lenses for reading where necessary. The MET is a four alternative forced-choice edge detection test which measures contrast sensitivity at around 3 c/° (Haymes and Chen, 2004). The lowest contrast edge correctly identified was recorded as the participant's contrast sensitivity in decibels (dB).

***Visual Fields.*** Automated static visual fields were measured binocularly using the Esterman protocol on the Humphrey Field Analyzer (HFAII M750, Carl Zeiss Meditec, Dublin, CA). Participants wore a working distance len correction as appropriate. The Esterman Efficiency Score (percentage of points seen) was recorded.

***Frisby Stereotest.*** Near stereopsis was assessed using the Frisby stereotest (Clement Larke International Ltd., London, UK). The test consists of three transparent plates of different thicknesses which present targets at different levels of depth disparity, ranging from 600 to 15 s of arc depending on the plate and testing distance. Testing was conducted using standard protocols and the level of stereoacuity presented in secs of arc.

***Useful Field of View Test (UFOV).*** The speed of visual information processing and visual-spatial attention were measured using the commercially available version of the Useful Field of View (UFOV) test, which is PC based and linked to a touch screen (17 in) for participant responses (Edwards et al., 2006). The first subtest requires the participant to identify a central target (outline of a car or truck) presented on a computer monitor within a central fixation box. The second subtest, which measures processing speed for a divided attention task, involves identification of the central target and simultaneous localization of a peripheral target (outline of a car), presented at one of eight radial locations. The third subtest included these two tasks as well as presentation of peripheral visual distractors that are triangles of the same size and luminance as the peripheral target car. Scores for each subtest are expressed as the display duration, in ms, at which the participant performed accurately on 75% of trials. Thus, scores for each subtest can range from 16 to 500 ms. The UFOV score represents the sum of the three subtest scores.

***Visual Motion perception threshold.*** Participants' threshold for perceiving the direction of central visual motion was measured using a computer-based random dot stimulus test (Wood et al., 2008). Participants were seated 3.2 m from the computer monitor under low light conditions. Computer generated random dot kinematograms were presented which consist of a pattern of randomly arranged high density black and white dots (or visual noise) a portion of which were coherently displaced in one direction (left, right, up or down). The direction and amount of displacement of the dots within the random dot display was varied successively over multiple trials such that the direction of movement became increasingly difficult to determine. The displacement threshold Dmin expressed as log of visual angle represented the smallest displacement for which the participant could determine the direction of motion.

#### Eye-disease

The presence of eye-disease was obtained via self-report. Participants indicated their history of eye disease by selecting responses to the following question: “Do/Did you have any of the following eye-related problems? (Tick all that apply).” Response options included cataract, glaucoma, macular degeneration, as well as the eye (right, left or both) affected.

#### Self-completed questionnaire

Participants completed a questionnaire that provided information on demographics: age, gender, education, source of income, accommodation, history of general medical conditions (stroke, TIA, diabetes, heart disease, arthritis, high blood pressure, peripheral vascular disease), medications, and level of assistance required for complex and self-care activities of daily living (ADL/IADLs) such as shopping, cooking, washing clothes, and dressing. Participants taking antidepressants were categorized based on responses to medication questions. Participants also provided data on whether they had undergone surgery for cataract extraction. Levels of anxiety and depression were measured using the 18 questions of the Goldberg Anxiety and Depression Scales (GADS) (Goldberg et al., 1988). The total score for each scale was used as the outcome variables in statistical analyses. Participants also undertook the MMSE (Folstein et al., 1975) as a screening measure for general cognitive impairment.

### Statistical analysis

All 662 participants had complete data for the following variables: age, gender, Mini-Mental status exam (MMSE) score, housing, self-reported myopia, presbyopia, visual field loss, congenital monocular blindness, as well as objective monocular measures of visual acuity under high contrast and low contrast. Data were missing for <5% of cases for years of education, income source, MET score, Frisby stereogram, and general medical conditions. Data for 10% of cases was missing for the visual motion threshold variable, and 39% of cases lacked data for the Esterman visual field score. In order to retain the full sample for analysis, missing variables were imputed using the Multiple Imputation in IBMSPSS Version 21. A missing value analysis indicated that data was not missing at random across the variables [Little's MCAR test: χ^2^_(185)_ = 702.53, *p* < 0.0001]. A Multiple Imputation was conducted using a model with all of the variables of interest as well as self-reported hearing impairment, body mass index, and general health conditions.

Separate analyses were conducted to examine anxiety and depression as correlates of vision function and eye disease. The total GAD score was also modeled separately as recent studies have questioned the capacity of GAD scales for differentiating depression from anxiety in older adults (Koloski et al., 2013). For each analysis, the respective GAD scale was used as a continuous outcome variable. Because the outcome variables were highly skewed and non-normally distributed we used a generalized linear model with the log link function and a Poisson distribution. The independent variables in each model included the self-reported eye disease categories, MET score, Frisby stereotest, average visual acuity (for the two eyes) for high contrast and average visual acuity under low contrast (logMAR units), Esterman visual field score, the motion sensitivity threshold (log degrees/second), and performance on the UFOV subtests. Covariates included in the model were age, gender, years of education, income source and accommodation type, MMSE score, the number of ADLs for which help was required, the number of general health conditions, self-reported myopia, long-sightedness, visual field loss and congenital monocular blindness, cataract surgery and antidepressant medications. The models also controlled for comorbid anxiety and depression. Goodness of fit statistics indicated that the Poisson with log link model produced a better model fit (Akaike Information Criterion: 1688.90) than the normal model with identity link (linear regression) (AIC: 2385.36). Bonferroni correction for multiple comparisons were conducted where relevant (α level = 0.0125).

## Results

The prevalence of any self-reported eye disease in the sample was 43.4%. Including those with comorbidity, the prevalence of self-reported cataract in our sample was 36.4%, glaucoma was 9.1%, and the prevalence of self-reported macular degeneration in the sample was 6.0%. Participants reporting comorbid AREDs accounted for 7.7% of the sample. Due to the small numbers of participants reporting both AMD and glaucoma (*n* = 4), cataract and AMD (*n* = 14) and triple comorbidity (*n* = 3) these groups were combined with those reporting both cataract and glaucoma (*n* = 30) to form the “Comorbid eye disease” category (*n* = 51).

The mean number of depressive symptoms on the GAD differed significantly across the self-reported eye disease categories –where AMD-only and the Comorbid group displayed nearly twice the mean number of depressive symptoms [*M*eans = 1.11 (1.76) and 0.96 (1.91), respectively] relative to the no eye disease group [*M*ean = 0.53 (1.16)] (Table 1). There was however, no significant difference in the mean number of anxiety symptoms across the eye disease categories. The GAD scales were originally validated in a young population (Goldberg et al., 1988) and recent data from Australian samples suggest that the average number of reported symptoms of depression and anxiety differ with gender and age cohort (Jorm et al., 2005). Based on these norms for older Australian adults (Jorm et al., 2005), above average levels of depression were relatively rare in our study sample (3.7% for no eye disease). Although this rate was higher in the AMD only group (10.5%), this was not a statistically reliable difference across the groups. Similarly, although the self-reported eye disease groups showed higher rates of above average anxiety (e.g., 10.5% in AMD and 11.8% in comorbid group vs. 8.3% in reference group) this was not a reliable difference (Table 1).

**Table 1:** **Demographic and health characteristics of the sample as a function of eye disease**.

	**No eye disease**	**Single eye disease**			**Co-morbid eye Diseases**	***p*†**
		**Cataract**	**Glaucoma**	**Macular degeneration**		
*N*	375	194	23	19	51	
Age (*SD*)	75.98 (4.36)	77.57 (4.5)	76.22 (2.89)	75.63 (4.25)	79.94 (4.91)	^**^
Sex (% males)	53.3%	42.3%	65.2%	42.1%	52.9%	
Years of education	11.54 (3.91)	11.43 (4.04)	11.78 (3.49)	11.63 (3.24)	11.06 (3.9)	
Mini-mental state exam	27.7 (1.79)	27.84 (1.67)	28.09 (1.7)	27.89 (1.73)	27.69 (1.6)	
**INCOME SOURCE**						
Pension	57.4%	55.0%	56.5%	42.1%	58.0%	
Self-funded retiree	39.3%	44.5%	43.5%	57.9%	40.0%	
Employed	3.3%	0.5%	0.0%	0.0%	2.0%	
**HOUSING TYPE**						
Own house or unit	93.6%	92.8%	91.3%	100.0%	90.0%	
Hostel/boarding/granny flat	6.1%	6.7%	8.7%	0.0%	0.0%	
Nursing home	0.3%	0.5%	0.0%	0.0%		
GAD depression	0.53 (1.16)	0.83 (1.55)	0.91 (1.41)	1.11 (1.76)	0.96 (1.91)	^*^
GAD anxiety	0.68 (1.36)	0.92 (1.6)	0.65 (1.46)	1.05 (2.17)	0.94 (1.76)	
Depression above average	3.70%	6.70%	4.30%	10.50%	7.80%	
Anxiety above average	8.30%	10.80%	8.70%	10.50%	11.8%	
Anti-depressant medication	8.50%	7.20%	4.30%	5.30%	9.80%	
General health—SF-36	74.48 (16.34)	67.82 (20.0)	74.52 (10.97)	71.42 (19.2)	67.18 (17.9)	^**^
Mental health—SF-36	84.63 (14.47)	81.56 (15.1)	80.17 (11.8)	82.95 (14.04)	80.71 (15.6)	
Physical functioning—SF-36	77.27 (19.44)	72.67 (20.98)	77.39 (14.84)	73.42 (22.24)	67.35 (22.3)	^**^
Stroke/TIA	6.4%	12.4%	26.1%	5.3%	13.7%	^**^
Diabetes	5.3%	7.2%	17.4%	5.3%	15.7%	^**^
Heart disease	21.1%	31.4%	17.4%	10.5%	31.4%	^*^
Hypertension	38.7%	46.9%	52.2%	47.4%	35.3%	
Requires assistance with ADLs/IADLs	4.3%	9.8%	4.3%	5.3%	9.8%	
Number of general health conditions	1.11 (0.05)	1.49 (0.08)	1.52 (0.27)	1.05 (0.19)	1.45 (1.12)	^**^
Cataract surgery	3.5%	57.2%	13.0%	10.5%	52.9%	^**^

* p < 0.05;

** p < 0.01;

Note: †omnibus ANOVA or Chi-squared tests.

Bivariate correlationss between the eye disease and vision variables and the depression and anxiety scales indicated that depressive symptoms were significantly correlated with eye disease category (Spearman's ρ = 0.102, *p* < 0.01), high contrast visual acuity (ρ = 0.212, *p* < 0.01), low contrast visual acuity (ρ = 0.235, *p* < 0.01) and visual motion sensitivity threshold (ρ = 0.211, *p* < 0.01). Anxiety symptoms were also significantly correlated with self-reported eye disease category (ρ = 0.08, *p* < 0.05), high contrast visual acuity (ρ = 0.205, *p* < 0.01), low contrast visual acuity (ρ = 0.215, *p* < 0.01) and visual motion sensitivity threshold (ρ = 0.190, *p* < 0.01).

A generalized linear model was used to examine eye disease and vision as correlates of depressive symptoms. The model controlled for age, gender, income source, accommodation type, the number of comorbid general health conditions, and level of assistance required for IADL/ADLs. Table 2 displays the unstandardized coefficients (*B*) and odds ratios (OR) for each independent variable pooled across the five imputed data sets. The main model effects indicated eye disease was significantly associated with depressive symptoms [Wald χ^2^_(5)_ = 13.52, *p* < 0.01]. Relative to the reference group reporting no eye disease, higher levels of depressive symptoms were associated with reporting cataract only [*B* = 0.33 (0.13), *p* < 0.0125; *OR* = 1.40 (CI 95%: 1.07–1.81)], and comorbid eye conditions [*B* = 0.60 (0.19), *p* < 0.01; *OR* = 1.82 (CI 95%:1.25–2.65)]. Those reporting AMD only also reported elevated levels of depressive symptoms, but this failed to reach significance after correction for multiple comparisons [*B* = 0.51 (0.25), *p* < 0.05; *OR* = 1.66 (CI 95%: 1.02–2.70)]. Independent of the type of eye disease, depressive symptoms were also associated with poor low contrast visual acuity [*B* = 0.04 (0.017), *p* < 0.05; *OR* = 1.06 (CI 95%: 1.0–1.08)].

**Table 2:** **Association between eye disease and visual function and depressive symptoms**.

**Variable**	***B*(*SE*)**	**CI 95%**	***OR***
Comorbid eye diseases	0.60 (0.19)	(0.23, 0.98)	1.82 (1.25, 2.65)^**^
AMD only	0.51 (0.25)	(0.02, 1.0)	1.66 (1.02, 2.70)^†^
Glaucoma only	0.37 (0.25)	(−0.12, 0.86)	1.44 (0.88, 2.36)
Cataract only	0.33 (0.13)	(0.07, 0.60)	1.40 (1.07, 1.81)^*^
Frisby stereogram	0 (0)	(0.003, −0.001)	1.00 (0.999, 1)
Melbourne Edge Test	0.02 (0.027)	(−0.03, 0.07)	1.01 (0.97, 1.05)
Esterman visual fields	0.003 (0.018)	(−0.04, 0.05)	1.00 (0.96, 1.05)
Visual acuity high contrast	0.069 (0.04)	(−0.016, 0.153)	1.07 (0.98, 1.17)
Visual acuity low contrast	0.04 (0.017)	(0.01, 0.07)	1.04 (1.0, 1.08)^*^
UFOV	0.000 (0.001)	(0.001, 0.006)	1.003 (1.001, 1.006)
Motion sensitivity threshold	0.06 (0.035)	(−0.01, 0.13)	1.06 (0.99, 1.14)

* p < 0.05;

** p < 0.01; Results Bonferroni corrected for multiple comparisons

† near significant.

Results for the vision and eye-disease as correlates of anxiety indicated that there was a main effect for eye disease [Wald χ^2^_(5)_ = 10.89, *p* < 0.05]. Table 3 shows that relative to those reporting no eye disease, self-reported cataract only [*B* = 0.34 (0.12), *p* < 0.01; *OR* = 1.41 (CI 95%: 1.11–1.79)] was significantly associated with depressive symptoms. Visual function also correlated with anxiety. Reduced low contrast visual acuity was associated with increased anxiety symptoms [*B* = 0.041 (0.018), *p* < 0.05; *OR* = 1.04 (CI 95%: 1.00–1.08)] as was edge contrast sensitivity [*B* = 0.06 (0.027), *p* < 0.05; *OR* = 1.06 (CI 95%: 1.01–1.12)] and visual motion perception [*B* = 0.16 (0.026), *p* < 0.001; *OR* = 1.17 (CI 95%: 1.11–1.23)].

**Table 3:** **Association between eye disease and visual function and anxiety symptoms**.

**Variable**	***B*(*SE*)**	**CI 95%**	***OR***
Comorbid eye diseases	0.35 (0.19)	(−0.06, 0.71)	1.42 (0.98, 2.04)
AMD only	0.46 (0.247)	(−0.023, 0.95)	1.59 (0.98, 2.58)
Glaucoma only	−0.03 (0.28)	(−0.58, 0.53)	0.93 (0.56, 1.70)
Cataract only	0.34 (0.12)	(0.10, 0.59)	1.41 (1.11, 1.79)^**^
Frisby stereogram	0 (0)	(0.003, −0.001)	1.00 (0.999, 1)
Melbourne Edge Test	0.06 (0.027)	(0.01, 0.11)	1.06 (1.01, 1.12)^*^
Esterman visual fields	−0.001 (0.021)	(−0.052, 0.05)	1.00 (0.95, 1.05)
Visual acuity high contrast	−0.027 (0.05)	(−0.14, 0.08)	0.97 (0.87, 1.09)
Visual acuity low contrast	0.041 (0.018)	(0.01, 0.08)	1.04 (1.00, 1.08)^*^
UFOV	0.00 (0.0003)	(0.00, 0.001)	1.00(1.00, 1.00)
Motion sensitivity threshold	0.16 (0.026)	(0.10, 0.21)	1.17 (1.11, 1.23)^**^

* p < 0.05;

** p < 0.01; Results Bonferroni corrected for multiple comparisons.

Analysis of the association between vision, eye disease, and total GAD score produced similar results. There was a main effect for eye disease [Wald χ^2^_(5)_ = 25.43, *p* < 0.001]. Table 4 shows that relative to those reporting no eye disease, high GAD scores were associated with self-reported cataract only [*B* = 0.36 (0.09), *p* < 0.001; *OR* = 1.43 (CI 95%: 1.20–1.71)], AMD only [*B* = 0.50 (0.18), *p* < 0.01; *OR* = 1.66 (CI 95%: 1.17–2.33)], and comorbid eye disease [*B* = 0.44 (0.13), *p* < 0.01; *OR* = 1.54 (CI 95%: 1.19–1.71)]. In addition, high GAD scores were associated with reduced edge contrast sensitivity [*B* = 0.06 (0.02), *p* < 0.01; *OR* = 1.06 (CI 95%: 1.02–1.10)], low contrast visual acuity [*B* = 0.05 (0.01), *p* < 0.01; *OR* = 1.04 (CI 95%: 1.02–1.09)] and motion sensitivity thresholds [*B* = 0.11 (0.02), *p* < 0.001; *OR* = 1.12 (CI 95%: 1.07–1.17)].

**Table 4:** **Association between eye disease and visual function and total GAD score**.

**Variable**	***B* (*SE*)**	**CI 95%**	***OR***
Comorbid eye diseases	0.44 (0.13)	(0.18–0.69)	1.54 (1.19, 2.00)^**^
AMD only	0.50 (0.18)	(0.16–0.85)	1.66 (1.17, 2.33)^**^
Glaucoma only	0.04 (0.19)	(−0.33–0.41)	1.04 (0.72, 1.51)
Cataract only	0.36 (0.09)	(0.18–0.54)	1.43 (1.20, 1.71)^**^
Frisby stereogram	0.00 (0.00)	(−0.001–0.00)	1.00 (0.99, 1.00)
Melbourne Edge Test	0.06 (0.02)	(0.02–0.09)	1.06 (1.02, 1.10)^**^
Esterman visual fields	0.004 (0.02)	(−0.05–0.05)	1.00 (0.96–1.05)
Visual acuity high contrast	0.04 (0.04)	(−0.06–0.12)	1.04 (0.95, 1.13)
Visual acuity low contrast	0.05 (0.01)	(0.02–0.09)	1.04 (1.02, 1.09)^**^
UFOV	0.00 (0.00)	(−0.00–0.001)	1.00 (0.999–1.00)
Motion sensitivity threshold	0.112 (0.02)	(0.07–0.16)	1.12 (1.07–1.17)^**^

** p < 0.01; Results Bonferroni corrected for multiple comparisons.

## Discussion

We examined the prevalence of self-reported comorbid eye disease, visual function status and depressive and anxiety symptoms in a community based sample of 662 older adults. Consistent with prior studies, we found that depressive symptoms were higher amongst participants reporting eye-disease relative to those who did not. Specific measures of vision function also significantly correlated with depressive symptoms, and this was apparent after controlling for age, socio-demographic factors, general health, anti-depressant medication, comorbid anxiety, treatment for cataracts, IADL/ADLs and self-reported eye disease.

In relation to self-reported eye disease, higher depressive symptoms were associated with reporting only cataract [*OR* = 1.40 (1.07–1.81)], comorbid eye disease [*OR* = 1.82 (1.25–2.65)] and a near-significant trend was apparent for AMD [*OR* = 1.66(1.02–2.70)]. These findings support previous reports of cataract as a predictor of depression (Fagerstrom, 1994; McGwin et al., 2003; Freeman et al., 2009) and additionally show that the relationship is independent of measures of visual function and is evident after adjusting for a range of health, demographic, treatment and socio-economic variables. Few studies have examined the relation between depression and multiple eye diseases. Our findings indicate individuals reporting multiple eye diseases are more likely to report higher levels of depressive symptoms independent of visual function and reporting a single eye disease. The results also revealed a trend for elevated levels of depression in those reporting AMD only—consistent with prior reports (Brody et al., 2001; Casten et al., 2004; Tolman et al., 2005; Mathew et al., 2011). The lack of any significant association between the presence of glaucoma and depressive symptoms in our data is also consistent with the few studies that have looked for and failed to find an association, after controlling for confounders (Wilson et al., 2002; Tosini et al., 2012). However, our data were only self-reported and glaucoma is known to be under-diagnosed in the population (Quigley, 1996).

In relation to symptoms of anxiety, higher levels of anxiety were associated with self-reported cataract alone [*OR* = 1.41(1.11–1.79)]. There is mixed evidence regarding the association between anxiety and eye-disease (Mabuchi et al., 2012; Tosini et al., 2012) or anxiety and visual impairment (Evans et al., 2007), and our study represents the only investigation of anxiety symptoms in relation to a range of eye disease through self-report as well as objective measures of visual function. Studies on the rate of anxiety and depression in cataract patients have largely focused around surgery, whereas the present findings relate to a non-surgerical context, suggesting that participants who are aware of having cataract may experience greater levels of generalized anxiety.

Our data reveal that objective indices of visual impairment and functional vision are associated with depressive symptoms and anxiety symptoms independent of self-reported eye disease. Specifically, poorer low contrast visual acuity increased the odds of depressive and anxious symptomatology by 4%. Poor edge contrast sensitivity also related to anxiety symptoms, and reduced motion sensitivity increased the odds of anxiety symptoms by 17%. Although small, these odds were statistically reliable. Several previous studies have reported a significant correlation between visual acuity and depression, independent of disease (Brody et al., 2001; Evans et al., 2007), however, most have used visual acuity at only one level of contrast (Brody et al., 2001; Rovner et al., 2006; Evans et al., 2007; Tosini et al., 2012), or visual acuity under both low and high contrast but not eye disease (Hayman et al., 2007). Nevertheless, most conclude that self-reported functional consequences of vision impairment may mediate the link between depression and objective measures of impairment (Brody et al., 2001; Rovner et al., 2006; Evans et al., 2007; Hayman et al., 2007; Freeman et al., 2009; Tosini et al., 2012). Although we did not use a measure of self-rated functional difficulties specific to vision as other studies had (e.g., VF-14) we found the need for assistance with IADLs/ADLs was more common in the eye-disease groups (4.3–13.3%) than amongst controls (4.3%), and our analysis adjusted for this. Further, we included objective measures of functional vision via the UFOV. This test is sensitive to age-related changes in vision, information processing speed and visual attention (Ball and Owsley, 1993), and is predictive of both falls risk (Vance et al., 2006) and driving capacity in older adults (Ball and Owsley, 1991). Interestingly, however, we found performance on the UFOV was not associated with depression or anxiety symptoms after adjusting for the other variables.

In the present study, we did not conduct a comprehensive cognitive assessment and used MMSE scores to control for significant cognitive impairment. Dementia is known to contribute to both depression and visual problems in older adults. Neuro-degeneration underlying dementia can affect the cortical and subcortical pathways of the visual system, and result in impaired visual function—including motion perception (Trick and Silverman, 1991; Iseri et al., 2006). In our study we excluded those with significant cognitive impairment, but may have retained individuals with mild cognitive impairment (MCI). Potential effects of MCI on visual function are not known, and post-mortem and neuroimaging data indicate the visual cortex is relatively spared of early neuro-pathological changes in MCI patients (Guillozet et al., 2003; Kempainen et al., 2007). Thus, based on current evidence, we expect that a direct effect of dementia-related neuropathology on visual problems would have been low. Nevertheless, depression is a neuropsychiatric symptom of both MCI and dementia (Lyketsos et al., 2002), thus, MCI may have independently contributed to depressive symptoms in some of our sample.

The relationship between specific vision measures and depressive and anxious symptomatology may reflect their effect on particular important activities such as reading, driving, and hobbies requiring close work (Tabrett and Latham, 2011). In our sample, poor low contrast visual acuity and reduced edge contrast sensitivity were associated with higher depression scores and also anxiety scores. Deficits in both of these domains could significantly impair daily function from activities such as reading through to mobility and driving. Further, reduced visual motion sensitivity may also impair judgment of environmental hazards and thereby increase anxiety related to mobility and falls. In addition to indirect effects on mood and anxiety via the impact of vision on function, the relationship may also include direct effects. Recent reports suggest that contrast sensitivity may be reduced in clinically depressed individuals, independent of any effect of mood on self-reported visual function (Bubl et al., 2013), and that even in the non-depressed, greater depressive symptoms are correlated with lower contrast sensitivity (Bubl et al., 2013). The authors further reported that contrast sensitivity normalizes with treatment of depression (Bubl et al., 2012). While the latter findings have not been substantiated in older adults with vision impairment, it suggests there may be biological pathways between mood and vision that are independent of eye-disease pathology and which may need to be considered when treating adults with comorbid depression and eye disease.

The present work has several limitations. A significant limitation is that eye-disease was obtained by self-report. Reported rates of glaucoma, cataract, and AMD are thus, biased by participant recall and awareness of often subtle changes in the early stages of the disease, particularly in the case of glaucoma (Wilson et al., 2002; Tosini et al., 2012; Voleti and Hubschman, 2013). We also did not have sufficient data to control for duration of diagnosis, which others have found may modify the relationship. As the study was cross-sectional in design, the reported relationships cannot reveal whether visual function and eye-disease predicted the development of depression and anxiety in this cohort, and longitudinal data is required to confirm such a causative relationship. Strengths of the study include the use of a relatively large community-based sample of participants with a range of eye diseases, making the findings more generalizable to the population. We also used objective measures of functional vision that are unlikely to be biased by low mood as would self-reported functional vision. Although the rate of eye disease was higher in the study sample relative to Australian population estimates (AIHW), the rates of depression and anxiety reported in our study are comparable to other population-based studies of eye-disease [4.6% in controls, 12% cataract, 15.1% for AMD (Evans et al., 2007); controls 3.0%, ARED 4.7% (Li et al., 2011)]; and anxiety [controls 4.3%; ARED 6.5% (Li et al., 2011)].

To conclude, the present study provides significant insights into comorbid depression and anxiety in older adults with self-reported eye-disease. The findings indicate that self-reported eye diseases as well as specific vision measures need to be considered when treating and managing older adults with eye disease. Primary care practitioners should consider eye disease and vision function in the context of depression and anxiety in older adults. The high contrast visual acuity chart available to most primary care practitioners do not assess low contrast visual acuity (Woods and Wood, 1995) that may affect everyday function and be associated with mood and anxiety in the older patient.

### Conflict of interest statement

The authors declare that the research was conducted in the absence of any commercial or financial relationships that could be construed as a potential conflict of interest.
